# Properties of biomass powders resulting from the fine comminution of lignocellulosic feedstocks by three types of ball-mill set-up

**DOI:** 10.12688/openreseurope.14017.1

**Published:** 2021-10-19

**Authors:** Rova Karine Rajaonarivony, Xavier Rouau, Charlène Fabre, Claire Mayer-Laigle

**Affiliations:** 1 IATE, Université de Montpellier, INRAE, Montpellier SupAgro, Montpellier, 34060, France

**Keywords:** Lignocellulosic biomass, Ball milling, Particle shape, Agglomeration, Powder flow properties

## Abstract

**Background:** Lignocellulosic biomass has many functionalities that hold huge potential for material, energy or chemistry applications. To support advanced applications, the biomass must be milled into ultrafine powder to increase reactivity. This milling unit operation needs to be fully mastered to deliver high-quality standard end-products. Here we studied the relationship between the characteristics of the starting lignocellulosic plant material and the properties of the resulting ultrafine powder in different ball-mill process routes.

**Methods:** Two lignocellulosic biomasses (pine bark and wheat straw) with contrasted compositional and mechanical properties were milled using three ball-mill set-ups delivering different balances of impact force and attrition force. The resulting powders were analysed for particle characteristics (size, agglomeration extent, shape) and powder flow properties (compressibility, cohesion) using a dynamic powder rheometer.

**Results**: Pine bark is more amenable to a fast particle size reduction than the fibrous wheat straw. The resulting pine bark powders appear less compressible but much more cohesive than the straw powders due to particle shape, density and composition factors. The mill set-up working by attrition as dominant mechanical force (vibratory ball mill) produced a mix of large, elongated particles and higher amounts of fines as it acts mainly by erosion, the resulting powder being more prone to agglomerate due to the abundance of fines. The mill set-up working by impact as dominant mechanical force (rotary ball mill) produced more evenly distributed particle sizes and shapes. The resulting powder is less prone to agglomerate due to a preferential fragmentation mechanism.

**Conclusions:** The attrition-dominant mill yields powders with dispersed particle sizes and shapes and the poorest flow properties, while the impact-dominant mill yields more agglomeration-prone powders. The mill set-up working with impact and attrition as concomitant mechanical forces (stirred ball mill) produces powders with better reactivity and flow properties compared to rotary and vibratory mills.

## Plain language summary

This research article describes the properties of two lignocellulosic biomasses milled into very fine powders (median diameter of 20 µm) in three different balls mills. The aim of this work was to see how the working principle of the milling devices shapes key properties of the milled powders for various end-use applications (3D-printed biocomposites, powder feedstock for lignocellulosic biofuel, source of platform molecules for green chemistry). In particular, we discuss the size, shape, agglomeration and flow properties of the powders produced in terms of their origin and comminution process route. The article then enlarges the discussion to the milling energy needed to produce the powders, in order to provide guidance on choosing the right milling technologies suitable for end-use applications.

## Introduction

There is substantial research directed at increasing the use of lignocellulosic feedstocks as a sustainable alternative to fossil resources for chemistry, energy and materials. The huge renewable stocks of lignocellulosic biomass sourced from agriculture and forestry/wood sectors are generally available in a format that has to be downsized and homogenized by dry grinding to make it processable. Furthermore, the growing technicality of emerging new applications such as smart 3D-printed materials ( Badouard *et al*., 2019; Ji *et al*., 2020; Mayer-Laigle *et al*., 2021a; Tao *et al*., 2017) or solid plant-sourced biofuels ( Kobayashi *et al*., 2008; Piriou *et al*., 2013; Stover *et al*., 2019) demands the use of ever finer lignocellulosic powders.

A major concern with ultrafine milling of plant materials is that it is hugely energy-intensive ( Karinkanta *et al*., 2018; Mayer-Laigle *et al*., 2018b; Wang *et al*., 2018). In a previous paper, Rajaonarivony *et al*. (2021) compared the dry milling efficiencies of three types of ball mills in terms of mechanical force delivered for the production of very fine powders (20-µm average particle size) from pine bark and wheat straw ( Rajaonarivony *et al*., 2021). They showed that impact and attrition processes resulted in different patterns of energy consumption producing powders with the same average particle size but different specific surface areas (SSA). First, the input energy was not transferred in the same way to the matter in all the devices, and second, the reaction of the plant matter to the mechanical forces differed according to type of biomass. A further concern is the individual (particle size, particle dispersion, particle shape) and bulk properties (degree of agglomeration, rheological response) of the ground powders. These properties will directly influence the quality of the end-products, and are a function of the milling process and the structural and functional properties of the original plant tissues, designed for protection and stress resistance ( Mayer-Laigle *et al*., 2018a; Oyedeji *et al*., 2020). Ultrafine milling of lignocellulosics to yield powders of appropriate and reproducible final characteristics is therefore a significant scientific and technical challenge.

The dimensional characteristics of ground powders are generally evaluated using a particle size distribution (PSD) curve ( Vaezi *et al*., 2013). For a same mean particle size, the PSD can vary depending on the nature of the biomass but also on the mechanical forces applied during the milling step ( Fabre *et al*., 2020). The width of the distribution, called span, and the presence of different peaks or modes in the curves evidence distinct responses to milling constraints in relation to the structure and tissue organization of the biomass material ( Mayer-Laigle *et al*., 2020; Rajaonarivony *et al*., 2019). Very fine powders of lignocellulosic biomass are more reactive than coarse powders as they offer considerably more available surfaces for physical and chemical reactions ( Karinkanta *et al*., 2018; Silva *et al*., 2012). The opening of new surface can change surface composition from the starting material as the fragmentation process can bring out chemical functions that had previously been buried in the bulk matter ( Zhang *et al*., 2019). This new reactivity can also affect the apparent PSD of the powders due to agglomeration phenomena ( Rajaonarivony *et al*., 2019). Indeed, the low density and surface properties of lignocellulosic matter mean that the force of inter-particle attractions may overcome the force of gravity for very small particles, resulting in the formation of clusters called agglomerates ( Gao *et al*., 2017; Opoczky, 1977; Rajaonarivony *et al*., 2019). As particle size decreases during milling, agglomeration processes may counteract fragmentation processes. This would translate into the PSD curves as a re-increase of the apparent mean particle size for long milling times and the emergence of a peak in the large particle range ( Blanc *et al*., 2020). These agglomerates, which decrease the overall SSA of the powder, can be partly dispersed by ultra-sound treatments. Nichols *et al.* (2002) distinguished soft and hard agglomerates, which correspond to easily dispersible agglomerates and irreversible agglomerates, respectively ( Nichols *et al*., 2002). Impact forces was reported to provoke more agglomeration with a larger share of hard agglomerates than attrition which results in only soft agglomeration ( Rajaonarivony *et al*., 2019).

Shape is another of the particle properties influenced by the nature of the biomass and the type of comminution mechanism generated by the milling device ( Guo *et al*., 2012). In general, coarse particles are irregularly shaped and more or less elongated depending on the fibrousness of the starting material. Over the course of milling, wheat straw and Douglas fir wood particles become more homogeneous and take on more spherical or parallelepiped shapes as their size decreases ( Silva & Rouau, 2011; Tannous *et al*., 2013). An attrition mechanism of comminution tends to yield a more elongated average shape than an impact mechanism ( Rajaonarivony *et al*., 2019).

The individual and bulk properties of milled particles (PSD, agglomeration status, shapes) are responsible for the macroscopic behaviour of the resulting powders ( Jan *et al*., 2018; Paulrud *et al*., 2002). Flow properties are especially important, as they dictate, in part, the end-use potential of the powders. Rheological properties can be approached by measuring the compressibility and cohesiveness of the powders ( Jan *et al*., 2018). In general, flowability is inversely related to compressibility and cohesiveness. The degree of compressibility comes from the deformability of the powder bed, which is related to particle shape and size distribution and its sensitivity to a normal stress ( Fu *et al*., 2012). Cohesiveness is linked to particle sizes and densities and their surface reactivity ( Freeman, 2007). Again, both these rheological indicators are expected to depend on the nature of the biomass and the way the powders were obtained.

However, despite the fact that the individual and bulk properties of milled particulates essentially govern the suitability of powder materials for target applications, there have been very few studies dealing with the determinant factors governing the final properties of a fine powder produced from lignocellulosic biomass. Addressing this gap would help to further promote the adoption of lignocellulosic biomass for various applications that require finely-tuned powder properties obtained in cost-efficient conditions. Here we performed an in-depth investigation of the influence of the mechanical forces generated by the three types of batch milling devices on the individual particle and bulk properties of ground powders from two contrasted biomasses, i.e. pine bark and wheat straw.

## Methods

The study took place between 2018 and 2019 in the PLANET facility run by the IATE joint research unit in Montpellier.

### Raw plant materials

This study used two source biomasses exhibiting contrasted mechanical properties: (i) bark from maritime pine ( *Pinus pinaster)* purchased in a local store (Botanic, Montpellier, France) and size-calibrated to 10–25 mm-length pieces, and (ii) wheat straw ( *Triticum aestivum*) harvested in 2015 at Saint-Gilles (France) and stored in 25-kg bales. They are hereafter referred to as starting materials. The moisture content of bark, which depends on storage conditions, was 35–40% when purchased and then reduced to below 15% by drying outdoors for 48h. The initial water content of wheat straw was around 11%. Moisture content was measured by weight loss after oven-drying for 2 h at 135°C.

### Sample preparation

Both lignocellulosic biomasses were first milled using a Retsch SM 300 cutting mill operating at 3,000 rpm equipped with a 2 mm-aperture sieving grid with manual feeding. Ground samples were then dried in an oven at 60°C to reach 3% moisture content before re-milling again with a Hosokawa Alpine UPZ 100 impact mill operating at 18,000 rpm and equipped with a 0.3 mm-aperture sieving grid and fed with a twin screw dosing system at a feed rate of 1.5 kg.h ^-1^. The resulting samples are named IM_bark and IM_straw, respectively. Before final comminution in the different ball mills, all samples were re-dried at 60°C to re-adjust moisture content to about 3%.

### Fine milling protocol

Three types of ball-mills were employed to finely mill the prepared samples: a rotary ball mill (RBM), a stirred ball mill (SBM), and a vibratory ball mill (VBM). These mills consist of a grinding chamber filled with different milling media (balls, beads, cylinders…) set in motion by either movement of the chamber or by a rotor. The biomass to grind is directly mixed with the milling media inside the chambers. After milling, the ground powders are separated from the milling media by dry screening. The different milling devices do not operate at exactly the same scale and so their milling chambers do not have exactly the same volumes. The milling parameters for each device, summarized in brief in Table 1, were defined based on the work of Rajaonarivony *et al*. (2021) ( Rajaonarivony *et al*., 2021) to enhance the mechanical forces generated by the different devices.

**Table 1.** Process parameters of the ball mills to yield powders with a 20-µm median particle size. RBM=rotary ball mill; SBM=stirred ball mill; VBM=vibratory ball mill.

	RBM	SBM	VBM
Description of the devices			
Model and supplier	Rotary ball mill, Faure, France	Stirred ball mill, custom-made, INRAe, France	Vibratory ball mill, DM10, Sweco, Belgium
Working principle	Biomass and ball-filled set in rotation by two rollers	A high-speed rotor drives the milling media mixed with the biomass to grind inside the milling chamber	Grinding chamber made in abrasion- resistant elastomer filled with the biomass and milling media, set in vibrating motion controlled by high-tensile steel springs
Chamber volume	2 L	3 L	36 L
Process parameters used in the study			
Milling media	3 kg of Ø25 - 20 -15-mm steel ball media distributed in a 1:1:1 ratio	5.7 kg of Ø6-mm steel beads	Blend of 25 kg of Ø12-mm ceramics balls and 25 kg of: Ø12-mm and 12-mm-length cylpebs
Speed/frequency	60 rpm	330 rpm	25 Hz
Mass of the sample to mill	0.2 kg	0.325 kg	1.0 kg
Mill fill before the milling operation (biomass + milling media)(%)			
Pine bark	46	49	42
Wheat straw	73	52	83
*Milling time to yield powder with a 20-µm median particle size*			
Pine bark	4.5 hours	0.36 hours	1.0 hour
Wheat straw	23.0 hours	1.6 hours	4.3 hours
Mechanical forces in the conditions of the study			
Dominant comminution mechanism	Impact	Balance of combined impact and attrition	Attrition

### Particle size analysis and agglomeration

PSD was measured using a Mastersizer 2000 laser diffraction granulometer (Malvern, UK). Several procedures were employed. Particle sizes were directly measured dry, which best describes the status of the powder regarding its application (protocol 1). In parallel, wet-method measurements were also carried out to evaluate the agglomeration status of the powder (protocol 2). The data was acquired using Malvern software version 5.40 (freeware downloadable on Malvern panalytical website). After, measurement data were saved as .txt files and processed with Microsoft Excel 2020. Alternatively, data can be processed using OpenOffice Calc (Apache open office).

***Protocol 1: Dry-method direct particle size measurements.*** Powders were fed into the device with a Sirocco 2000 feed hopper (feed rate: set to 50% of maximum at 3-bar air pressure). Particle distribution, median particle size (d50) and specific surface area (SSA) were determined based on Fraunhofer theory ( de Boer *et al*., 1987). We also calculated the size span, which is an expression of the width of the distribution, based on Equation (1).

$$\;span=\frac{d_{90}-d_{10}}{d_{50}}\;\text{eq.}\;1$$

***Protocol 2: Wet-method PSD and agglomeration measurements.*** PSD was also measured by wet method to allow the use of ultra-sound to de-agglomerate the powders and evaluate agglomeration status. First, the PSD of the powders and their SSA were measured in ethanol (96% v/v) in the Mastersizer 2000 laser diffraction granulometer (Malvern, UK) equipped with a Hydro 2000S ultrasonic system to disperse the agglomerates. Data were processed following Mie theory using the refractive index of wood sawdust (1.53) ( de Boer *et al*., 1987). The powder was then deagglomerated using either the Mastersizer-embedded Hydro 2000S ultrasonic deagglomeration system (SSA __LG_) or a more powerful external ultrasonic probe (SSA __EP_), and the PSD was determined using the Mastersizer granulometer. All measurements were carried out in duplicate.

Using the Mastersizer ultrasonic system, a 3-min sonication at maximum probe power (75 W) was applied to a suspension of approximately 0.1 g of powder in 200 mL of ethanol previously dispersed in the Hydro 2000S system. The suspension was then stirred at 3000 rpm for 5 minutes to remove any bubbles prior to measurement of SSA_ _LG_. Using the external power ultrasonic probe, a suspension of 0.1 g of powder in 200 mL of ethanol was stirred with a magnetic agitator then sonicated for 5 min using a ¼” Qsonic Q700 ultrasound Microtip probe (Qsonica, USA) at maximum power (130 W). The dispersion was then fed into the Hydro 2000S system connected to the Malvern Mastersizer, and particle size measurements were performed after applying an additional 30 s burst of sonication using the granulometer ultrasound probe at maximum probe power (75 W).

However, we observed a partial dissolution of the ultrafine pine bark powder in ethanol (particles below 2 µm), with quantity of dissolved particles depending on both particle size and measurement time. To limit the bias in the interpretation of the results, we considered 2 µm as the size-limit resolution of this methodology, and recalculated the PSDs and specific surface areas taking into account only the particles larger than 2 µm. The agglomeration data is therefore underestimated, but the results can still be discussed for purposes of comparative analysis.

SSA was calculated according to Equation (2) ( Blanc *et al*., 2020) and expressed in m ^2^.mm ^-3^ to accommodate differences in density.

$$\;SSA=3*\sum_{i}\frac{\alpha_{i}}{R_{i}}\;\text{eq.}\;2$$

where i is the index grading class (between 2 µm and 2000 μm), αi is volume fraction, and Ri is average radius of particles in size class i.

We wrote SSA, SSA_ _LG_ and SSA_ _EP_ to denote specific surface areas measured prior to deagglomeration treatment, after deagglomeration using the Mastersizer ultrasonic probe, and after ultrasonic treatment using the external probe, respectively.

Hard and soft agglomeration were thus determined as follows:

$$Soft\;agglomeration=\frac{SSA_{-LG}-SSA}{SSA\_EP}\;\text{eq.}\;3$$

$$Hard\;agglomeration=\frac{SSA_{EP}-SSA}{SSA\_EP}\;\text{eq.}\;4$$

Agglomeration was also examined by analyzing shifts in specific particle-size populations through different deagglomeration procedures. The size classes studied were 2 µm–5 μm, 5 µm–20 μm, 20 µm–80 μm, and >80 μm. We used 5 µm and 80 μm as thresholds as they correspond to 4 times below and 4 times above the median particle size of 20 μm, respectively.

The method proposed here is inspired by population balance modeling ( Gil *et al*., 2015). From each particle-size distribution, i.e. not deagglomerated (ND), deagglomerated by laser granulometer (LG), and deagglomerated by the external probe (EP), we determined the volume weight (volume proportion) of particle populations ( *V _i to j_*) by summing the volume weight of each grading class ( *V _x_*) in the considered range (size in between i and j, with i = 2, 5, 20, 80 and j = 5, 20, 80, 200). As there were no particles >200 μm in the ultrafine powders, the classes > 200 µm were not considered.

$$\;V_{i\_j}=\sum_{x=i}^{x=j}V_{x}\;\text{eq.}\;5$$

We then determined the volume weight difference of each population to evaluate the particle-size shift from one class to another when the deagglomeration procedure is applied, which indirectly gives us access to the sizes of the agglomerates and their constitutive particles.

### Particle shape measurement

Particle shapes were evaluated using a Morphologi 4 automated morphological imaging and particle characterization system (Malvern, UK). First, a defined volume of powder was dry-dispersed on a glass plate using the Morphologi 4 system’s integral sample dispersion unit. The sample volume defined is dependent on particle size and has to allow a representative view of the sample without overlapping particles. Pictures of the dispersed particles were then taken by the device with one or several selected optical lenses. Due to differences in lens depths, the surface of the plate analysed has to fit with the selected optical lens to get accurate definition of all particles. In this study, we used ×5, ×10 and ×20 magnifications with analysis area zones of 700 mm ^2^, 500 mm ^2^ and 50 mm ^2^, respectively. In order to analyse at least 100,000 particles per sample, we defined three sampling zones per plate, and the results were merged to determine the shape factors.

In practice, this study used two different protocols according to particle size of the powders.

***Protocol 1: Powders produced via a combination of cutting mill followed by impact mill.*** Volumes of 11 mm ^3^ (IM_bark) and 15 mm ^3 ^(IM_straw) were dispersed with an air pressure of 1 bar and an injection time of 20 ms (low-pressure dispersion) to obtain a homogeneous dispersion on the plate, despite the strong morphological disparity. The volumes of dispersed powder were determined based on preliminary tests. Particles were analyzed at ×5 magnification. In the analysis, only particles of diameter > 4.5 µm were considered in the analysis, as the selected magnification gives too few pixels to obtain precise shape factors for smaller particles. In addition, to accurately define all the particles, images were acquired by z-stacking two images. This method involves taking images at two different levels (with the height difference between the two levels being equal to the depth of field) and rebuilding a sharp image of each particle, even in the case of polydisperse particle sizes and thick particles.

***Protocol 2: Fine powders produced by the RBM, SBM and VBM.*** 5 mm ^3^ of powder was dispersed using an air pressure of 4 bars and an injection time of 10 ms (high-pressure dispersion) to overcome the inter-particle forces, which are stronger for smaller particle sizes. Fine biomass particles can be slightly transparent, which makes it very difficult to get a satisfactory focus for both small and large particles with the same optical lenses. To overcome this difficulty, particles with a diameter in the 2 µm–20 µm range were analysed with ×20 magnification and particles > 20 µm were analysed with ×10 magnification. As the size of 20 µm corresponds to the median particle size targeted for the powders in this study, global distributions were built by post-processing, considering that particles below 20 µm and particles above 20 µm each accounted for 50% of the total. As in protocol 1, images were acquired by z-stacking two images.

For both protocols, the following shape factors were extracted from the acquired images: diameter, elongation ( Equation (6)), and convexity ( Equation (7)):

$$\;Elongation=1-\frac{W}{L}\;\text{eq.}\;6$$

where W and L are the maximum width and length of the particles, respectively. An elongation of 0 corresponds to a perfectively round or cubic particle, and an elongation of 1 corresponds to an infinitely elongated particle (with no width).

$$Convexity=\frac{convex\;hull\;perimeter}{perimeter\;of\;the\;particle}\;\text{eq.}\;7$$

The convex hull is the smallest convex polygon that contains all the vertex of the particle. A perfectly smooth particle will have a convexity of 1. The convexity factor thus gives an indication of the fractal geometry of the particle surface. In the following, convexity is only discussed based on its median value. For elongation and diameter, we considered the volume distribution of the powder and post-processed the data using Matlab software (Matlab R2017b- MathWorks) to build 3D plots combining both the elongation and the diameter volume distribution. Alternatively, the data can be process using Julia.

### Microscopy

Samples were imaged by scanning electron microscopy (SEM). Samples were stuck onto carbon adhesive tape and observed directly, without metallization, using a benchtop Phenom ProX scanning electron microscope (Phenom World, The Netherlands) with an acceleration voltage of 10 kV in image mode and a backscattered electron detector.

### Powder flow properties

The flow properties of the powder were quantified using the Carr index and the compressibility and the cohesion tests of the FT4 powder rheometer (Freeman Technology, UK) ( Freeman, 2007). Before each measurement, powders were oven-dried at 60°C for 24 hours to overcome the influence of water adsorbed onto the surface of the particles.

Carr index: First, apparent powder density ( *ρ _a_*) was determined by simply filling and weighing a 250-ml glass cylinder and measuring packed density ( *ρ _p_*) by submitting this cylinder to 150 stroke cycles in a Densi-tap device (Matec, France). Carr index (Ci) ( Carr, 1965) was then calculated according to Equation (8):

$$\;Ci=\frac{\rho_{p}-\rho_{a}}{\rho_{p}}\;\text{eq.}\;8$$

Compressibility tested by the FT4: The compressibility of a powder is the ability of a bed of powder to reduce in volume under the action of normal mechanical constraints. Measurements were conducted using the FT4’s 85-ml vessel. After filling the vessel with the powder, the powder bed was standardized with a conditioning step before each measurement consisting in a rotation of the blade through the entire height of the powder bed. After the conditioning step, the powder was run through increasing normal stresses from 0.5 kPa to 15 kPa (0.5, 1, 2, 4, 6, 8, 10, 12, 15 kPa). For each stress applied, the height of the powder bed was measured and the percentage of compressibility was deduced (Cp). Percent compression was calculated according to Equation (9), where Vi is initial volume of the powder bed in the vessel and V _15kPa_ is volume of powder after applying a 15 kPa normal stress.

$$\;Cp=\frac{V_{i}-V_{15kPa}}{V_{i}}\;x\;100\;\text{eq.}\;9$$

Cohesion: The cohesion of the powder is determined from the shear test performed on the FT4. After filling the vessel (85 mL) with the powder, the powder bed was standardized with the conditioning step previously described, then consolidated under a normal stress of 3 kPa. The powder bed was then further consolidated with 5 increasing normal stresses from 1 to 2 kPa (1, 1.3, 1.5, 1.7, 2 kPa) and the torque required to shear the powder bed was measured. In this work, shear stress was linearly fitted using the Mohr–Coulomb failure criterion, where *τ* is shear stress (kPa), F is static friction coefficient, *σ* is normal stress (kPa) and C is cohesion (kPa). The cohesion value (Co) is the point where the regression line intersects the y axis ( Equation (10)):

τ(σ)=Co+F.σeq.10

## Results & discussion

The primary purpose of milling is to reduce particle size. However, the efficiency of the milling process depends above all on the properties of the feed material. In-depth knowledge of the feed particle characteristics is therefore essential to understand how the fine comminution step will perform. In the following, we discuss the particle sizes and shapes of the ultrafine 20-µm-median-particle-size powders and their source feed powders produced by the cutting-mill and impact-mill steps.

### Influence of ultrafine milling on particle size distribution

***Particle sizes of the feed powders.*** The maritime pine bark and wheat straw starting materials were purchased as amounts of cm-range pieces (10–25-mm chips for pine bark, 5–30-cm hollow stalks with leaves for wheat straw). These materials cannot be milled directly with good efficiency in ball mills designed for ultrafine powder manufacturing. Pre-milling steps are required to reduce the particle size to a suitable input format (generally below ~500 µm average particle diameter). This was achieved here by sequential processing, first in a cutting mill for coarse grinding (from cm down to mm range) then in an impact mill for intermediate grinding (from mm down to hundreds-of-µm range).

**Figure 1.**
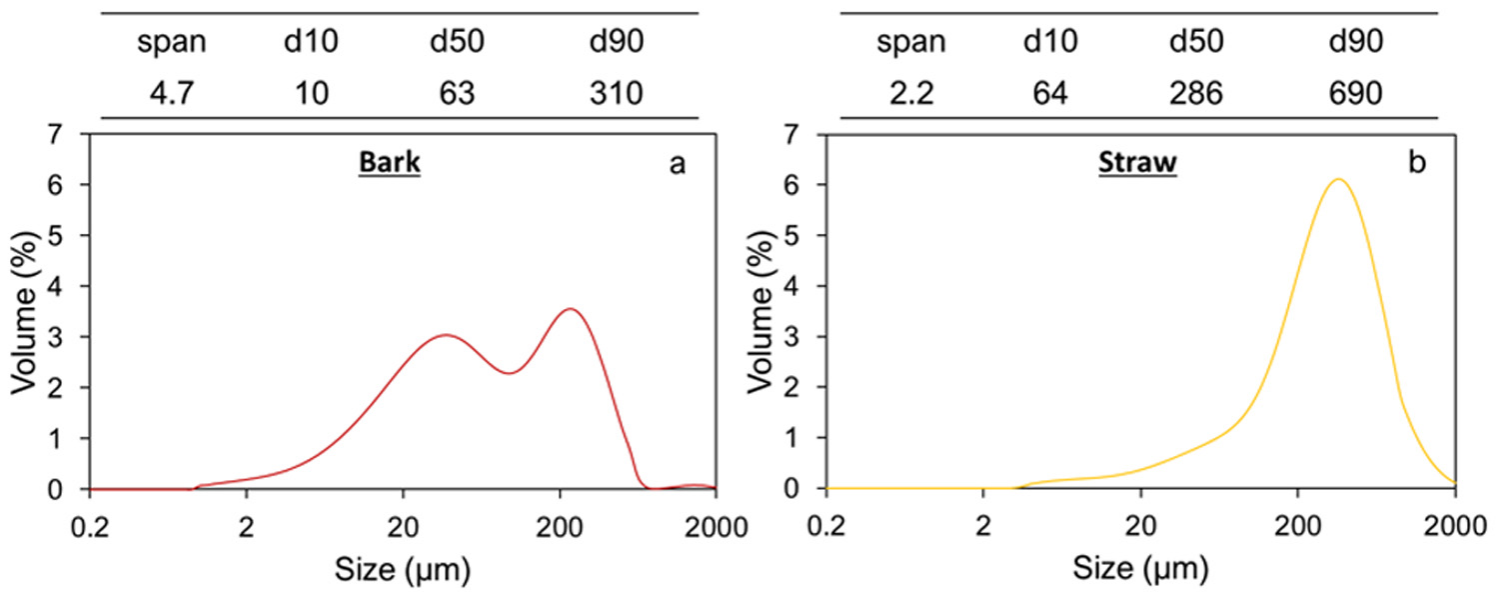
Particle size indicators and distributions of bark (a) and straw (b) powders after impact milling.

The median size of bark and straw powders obtained after the first milling step (cutting milling) were 320 µm and 600 µm, respectively ( Mayer-Laigle *et al.*, 2021b). Given that their initial formats were not really equivalent, this points to better grindability of bark than straw. The PSD and the main size indicators of the powders resulting from the second preparation step, i.e. impact-milling, are reported in Figure 1. These samples, which correspond to the input material to feed the ball mills, are called IM_bark and IM_straw, respectively.

This figure shows differences in the particle distribution pattern between the bark and straw powders. The bark powder exhibits a bimodal distribution with two peaks of similar height corresponding to particle size populations centred on 40 µm and 225 µm, respectively. The straw powder distribution has a unimodal profile with a median particle size of 280 µm, although a small peak drag is visible towards the small particle range (30–40 µm). Overall, after being subject to the same pre-milling process, the pine powder is much finer than the straw powder, with a 4.5 smaller d50, and a d90 around 300 µm *versus* 700 µm. However, the spread of the distribution, as indicated by the span value, is significantly narrower for straw than for bark.

Pine bark thus shows more heterogeneity than wheat straw at this level of mechanical deconstruction. Given the general organization of plants, it can be assumed that particle populations below the 50-µm range are mostly composed of small clusters of cells, single cells or cell debris, and that particle populations in the upper range (hundreds of µm) are mostly composed of tissue fragments. Bark is a multi-layered material composed of three main tissues, i.e. phloem, periderm, and rhytidome ( Nunes *et al*., 1996), that differ in cellular composition, cell shapes and mechanical properties. Data on *Pinus pinaster* bark is scarce, but studies on related species ( *Pseudotsuga menziesii* or Douglas fir ( Ferreira *et al*., 2016; Trivelato *et al*., 2016); *Pinus pinea* ( Miranda *et al*., 2017)) have shown that grinding coniferous tree bark also results in several fractions, with the finest fraction originating from weakly-lignified and fragile parenchymatous tissues and the coarsest fraction from highly-lignified and tough sclerenchymatous tissues. Wheat straw is a complex material comprising mostly hollow stalks structured as a succession of nodes and internodes ( Harper & Lynch, 1981). The stalks are composed of a parenchyma containing vascular bundles surrounded by an epidermis rich in cellulose, wax and silica, which led the stems resistance, elasto-plasticity and hydrophobicity. Straw is a fibrous material that contains elongated cellulose fibres in the 5–10-µm size-range, oriented according to the length axis of the stem ( Yu *et al*., 2008). Straw internodes thus exhibit high deformability and longitudinal toughness, but poor transversal resistance. The scale of mechanical constraints and level of reduction in the impact mill make it impossible to reveal distinct particle populations with the straw at this step, unlike for pine bark.

Due to differences in microstructure and particle sizes and shapes, after the two pre-milling steps, the apparent density of bark powder (380 kg.m ^-3^) was twice that of straw (190 kg.m ^-3^). This difference will go on to affect further processing, because at identical weights of powder, the volume of the straw was higher than that of the bark. The values of the corresponding packed densities (480 kg.m ^-3^ for bark and 210 kg.m ^-3^ for straw) indicate that bark powder packs more than straw.

**Figure 2.**
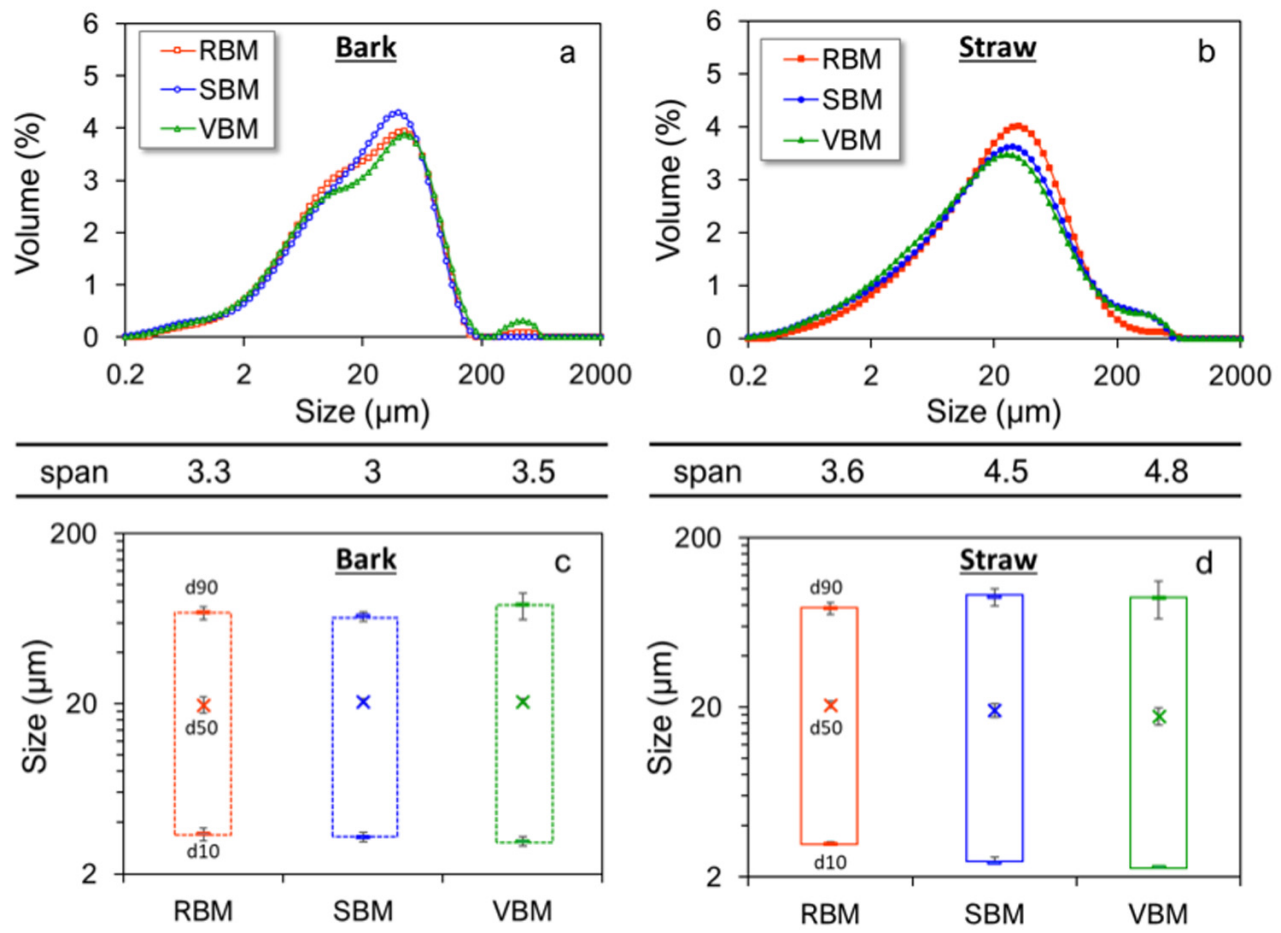
Particle size indicators and distributions of fine bark (a, c) and straw (b, d) powders. Particle size distribution, span and size indicators (d10, d50, d90) for bark ( **a**, **c**) and straw ( **b**, **d**) milled in RBM (rotary ball mill), SBM (stirred ball mill) and VBM (vibratory ball mill).

***Particle size distribution of the ultrafine ground powders.*** Figure 2 shows the PSD of 20-µm-median-particle-size bark and straw powders obtained with the different ball mills. Ultrafine milling tended to smooth the bimodal distribution observed in IM_bark. For the SBM, PSD became unimodal. RBM and VBM powders still had two visible peaks, but they were much less pronounced than in IM_bark. The ultrafine milling step also reduced the span value from 4.7 to 3.3, suggesting that ultrafine milling led to a homogenization of the bark powder.

In contrast, the PSD profiles of straw remained unchanged by ultrafine milling, but the span value increased significantly from 3.6 to 4.8. The ultrafine milling step thus reveals a heterogeneity for the straw that was not visible at the upper size scale. The structural heterogeneity of the two biomasses is thus expressed at different scales.

In the case of bark, the size indicators d10, d50 and d90 remained very similar, whichever mill was used. However, in the case of straw, there were clearer differences between milling devices. The RBM led to similar d10 and d90 values both for bark and straw. However, at similar d50, the d10 obtained in SBM and RBM were lower for the straw than those obtained for the bark and the d90 higher leading to higher span values (reflecting the spread of the PSD). Thus, it seems that the milling devices generating attrition as dominant mechanical forces are more sensitive to the histological structure of the materials than the impact-mechanism milling device. The greater d90 value observed in the case of straw may be related to the greater anisotropy of straw due to the presence of long and elastic fibres, as previously discussed ( Du & Wang, 2016). The attrition comminution mechanism, which erodes the particles by frictional and shear forces, progressively dissociated small pieces from the surface of the particles without causing deep damage to the structure of the fibres. Note that the d10 values obtained in RBM and VBM were smaller for the straw than for the bark, which generally connects to the better grindability of the raw straw material, as discussed above. However, this may be also attributed to a larger extent of agglomeration phenomena in the case of bark particles, as described in previous work ( Rajaonarivony *et al*., 2019; Rajaonarivony *et al*., 2021). We clarify this point further down in a section on in-depth granulometric analysis of the different classes of particles.

### Particle shape

***Particle shape of feed particles.*** The shapes of the feed particles were studied by image analysis. Equivalent spherical diameter, elongation, and convexity were recorded from the images. Equivalent spherical diameter corresponds to the diameter of a sphere whose diffraction surface is identical to the diffraction surface of the real particle. Elongation is the ratio of the difference between the two main dimensions of the particle (length and width) divided by length of the particle. Particles with an elongation value close to 1 are thus very thin in comparison to their length, whereas spherical or cubic particles will have an elongation value close to 0. Convexity reflects the surface state of the particles, in particular their roughness, and is calculated by dividing the perimeter of a totally smooth particle (of the same surface) by its actual measured perimeter. A totally smooth particle will have a convexity of 1, whereas a particle with a fractal surface will have a convexity closer to 0.

Median equivalent spherical diameter values deviated by about 20% from the values measured by laser diffraction granulometry. This could be attributed to the fact that (i) the image analysis was performed in two dimensions whereas laser diffraction measures particles in a flow in all three dimensions, and (ii) only the particles >4.5 μm were considered in the calculation of median particle size by image analysis due to the lens performance limits.

**Figure 3.**
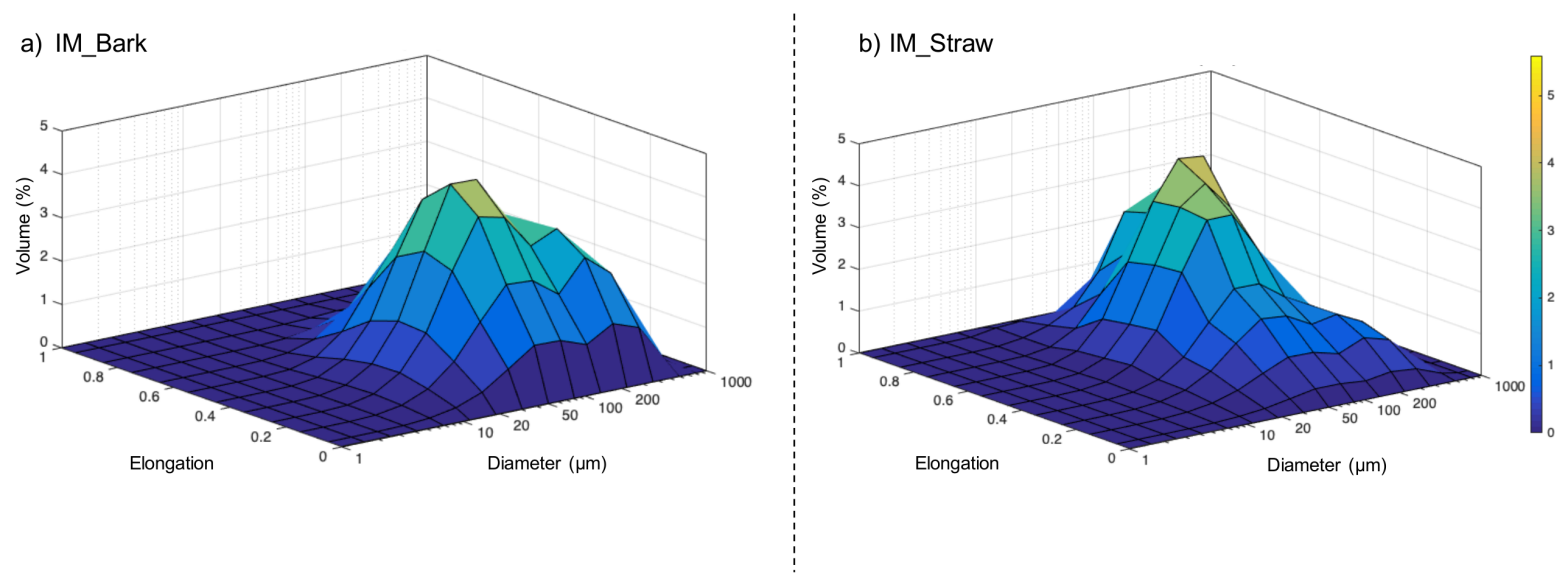
3D-plot diagram mapping the distribution of size particle *vs* elongation in the feed powders. 3D-plot diagram mapping the distribution of equivalent spherical diameter vs elongation in the feed powders of IM_bark ( **a**) and IM_straw ( **b**).

The median elongations obtained for the particles of IM_bark and IM_straw were 0.33 and 0.55, respectively. This means that a median bark particle has a width equal to ⅔ of its length and a median straw particle has a width that is ½ of its length. However, the size of the raw particles is distributed over more than a centile and the mean value may not reflect the shape factors for the different populations of particles. In particular, in previous work ( Rajaonarivony *et al*., 2019), Rajaonarivony *et al*. demonstrated that the shape factor is a function of particles size—smaller particles tend to have more regular shapes. Figure 3 gives 3D-plots of elongation for different particle-size classes of bark ( Figure 3a) and straw ( Figure 3b). For the bark, the profile of the PSD (diameter axis) is similar to the profile from laser diffraction (section 1.1) with a pronounced bimodal distribution, but the particle elongations are relatively homogenous, ranging between 0 and 0.6 whatever their diameter. In contrast, for the straw, the PSD is more narrowly spread around the median particle size, as discussed above, but the elongation is spread between 0.1 and 0.95, showing a high disparity in shapes related to the fibrous structure of the straw feed material.

The convexity values were very similar between bark and straw, suggesting that this indicator is not very sensitive to the difference in histological structure but may be more affected by the milling process.

**Figure 4.**
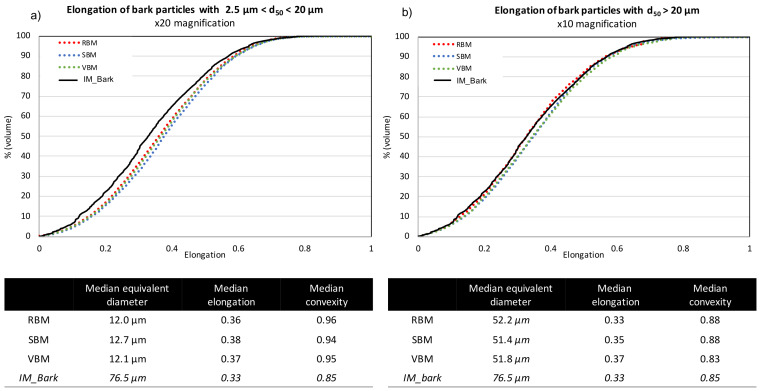
Cumulative distribution of elongation for bark particles obtained in RBM, SBM and VBM processes. Cumulative distribution of elongation for bark particles obtained in RBM (rotary ball mill), SBM (stirred ball mill) and VBM (vibratory ball mill) processes and median values for particle size, elongation and convexity. ( **a**): fine particles with 2 µm < d50 < 20 µm; ( **b**): larger particles with d50 > 20µm.

**Figure 5.**
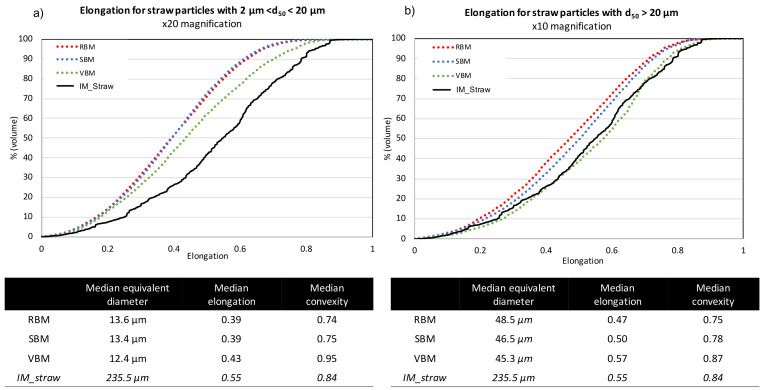
Cumulative distribution of elongation for straw particles obtained in RBM, SBM and VBM processes. Cumulative distribution of elongation for straw particles obtained in RBM (rotary ball mill), SBM (stirred ball mill) and VBM (vibratory ball mill) and median values for particle size, elongation and convexity. ( **a**): fine particles with 2 µm < d50 < 20 µm; ( **b**): larger particles with d50 > 20 µm.

***Particle shape of ultrafine powders.*** Figure 4 (for bark powder) and Figure 5 (for straw powder) show the cumulative distributions of the particle elongations from the three milling devices. Due to the dispersion of the powders, in order to get an accurate definition of all particles, they were analysed with two different optical lenses: ×20 magnification for particles < 20 µm according to protocol 1 (see Material and methods) ( Figure 4a and Figure 5a) and ×10 magnification for particles > 20 µm according to protocol 2 ( Figure 4b and Figure 5b). Furthermore, this division evidenced differences in behaviour between the smaller and coarser particles. Interestingly, for a given material *—*bark or straw—the measured median particle sizes were similar whatever the milling device, confirming laser diffraction granulometry observations that although there are small differences between the particle size indicators (d10, d90 and SPAN) in the case of straw, the overall PSDs are very similar.

For bark particles ( Figure 4), the different shape indicators are also similar across all milling devices. The median convexity of coarse > 20 µm particles is quasi-equal to the convexity of the input bark particles ( Figure 4b), whereas the median convexity of < 20 µm particles is close to 1, suggesting that the smallest particles of the bark powder had a practically smooth surface ( Figure 4a). The cumulative elongation distributions evidence that the different milling devices produced very few differences in terms of morphology for bark powders. All the distributions collapse together, whether for the smallest or the coarsest particles. Thus, for bark powder, the different comminution mechanisms imparted by the different devices do not induce significant differences in terms of particle shapes. Note, however, that the smallest particles are more elongated than the input bark particles, as all cumulative distributions were below those of the IM_bark. The different milling processes tend to slightly elongate small particles, as if they stripped the materials into small needles.

Straw particles ( Figure 5) showed far more pronounced differences between the shape factors of powders from the different milling devices. For the smallest particles (< 20 µm., Figure 5a), the cumulative distributions for RBM and SBM devices were very close, with smaller and median elongation significantly decreasing to reach a value of 0.39, which was similar to the elongation value for the small particles of bark. In VBM, a decrease of the elongation is also observed with decreasing size, although the difference with the IM_straw was weaker than with the RBM and SBM devices. For the coarsest particles (> 20 µm., Figure 5b), the cumulative elongation distributions increased from RBM to SBM to VBM. Interestingly, the milling equipment that is more attrition-dominant produces particles that are more elongated and closer to the shape of IM_straw particles. Note that the VBM distribution merges with the IM_straw distribution, suggesting that the VBM process induces a homothetic comminution of particles through its dominant mechanism of attrition.

**Figure 6.**
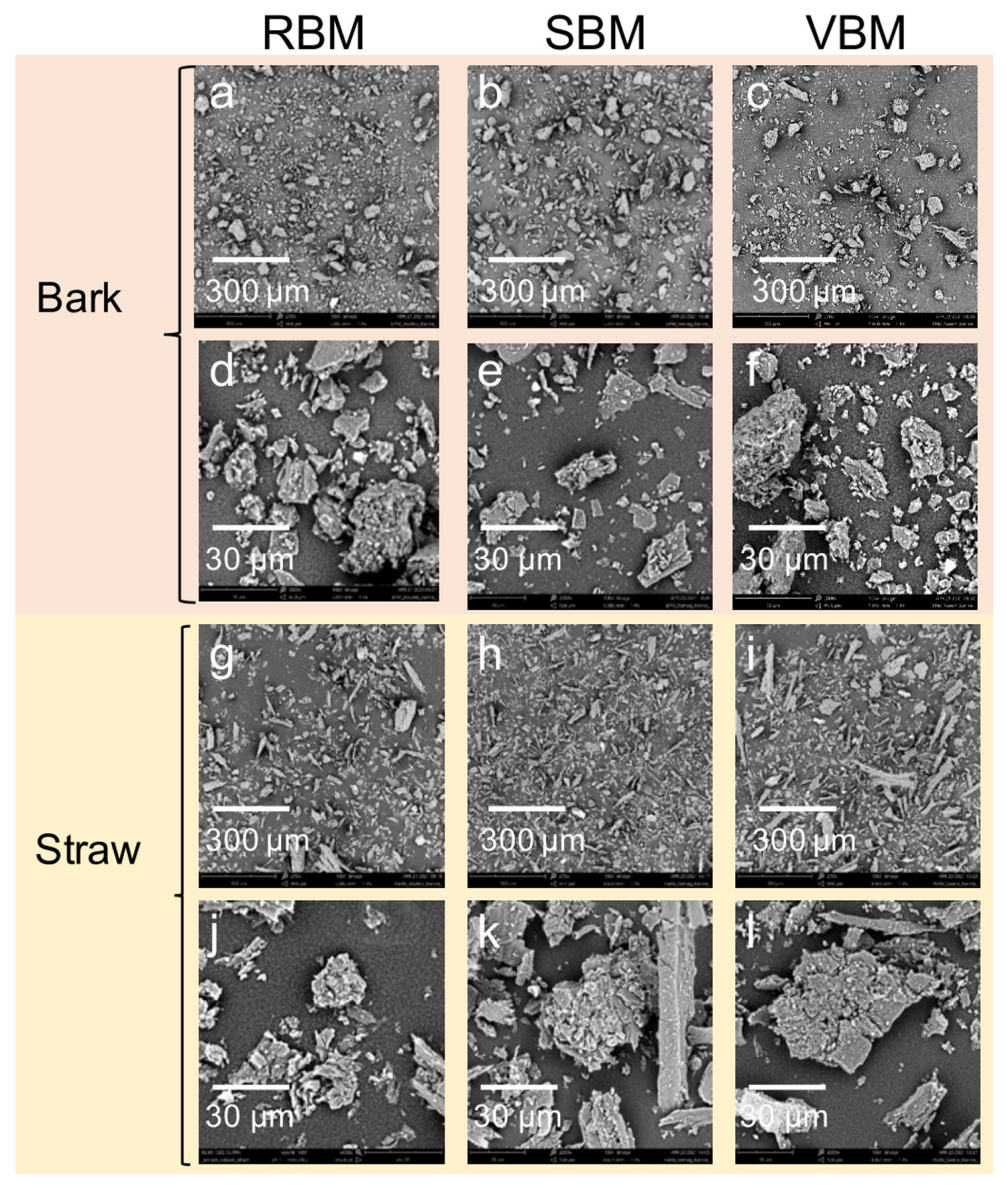
SEM micrographs of bark and straw 20 µm-centred powders at two magnification scales. Raw unedited SEM micrographs of bark and straw 20 µm-centred powders at two magnification scales **a**. RBM_bark x270; **b**. SBM_bark x270; **c**. VBM_bark x270; **d**. RBM_bark x2000; **e**. SBM_bark x2000; **f**. VBM_bark x2000; **g**. RBM_straw x270; **h**. SBM_straw x270; **i**. VBM_straw x270; **j**. RBM_straw x2000; **k**. SBM_straw x2000; **l**. VBM_straw x2000.

Figure 6 presents micrographs of the six ultrafine powders using two magnification scales in order to illustrate (i) the general distribution of particle shapes in each powder (×270 magnification) and (ii) the shape and structure of the particles (×2000 magnification).

In Figures 6g, 6h and 6I, the straw particles appear much more elongated and the distribution is more spread out than in Figures 6a, 6b and 6c that show bark particles. Furthermore, Figure 6j shows that straw particles from the VBM appear much more elongated than in Figures 6g and 6h that correspond to particles resulting from straw milling in the RBM and SBM, respectively.

Figures 6d, 6e and 6f and 6j, 6k and 6l clearly show that the smallest particles stick to the surface of coarser particles, suggesting an agglomeration phenomenon that is extensively investigated in the following section.

### Agglomeration phenomena

When particle size decreases, the inter-particle forces may overcome the force of gravity, enabling agglomeration phenomena to occur. Small particles aggregate together or stick to the surface of coarser ones, creating increasingly coarse particles. Agglomeration may be reinforced by particle size and shape and surface composition. Agglomerates can fall into two forms: (i) soft agglomerates, which are relatively weak agglomerates that can be easily dispersed, and (ii) hard agglomerates, which result from strong inter-particulate bonding (as chemical or cooperative hydrogen bonds), causing the particles to become tightly bound and difficult to re-disperse ( Hu *et al*., 2012). Agglomeration reduces milling efficiency and modifies the properties of milled end-products. We previously developed an indirect method for quantifying both soft and hard agglomeration based on the specific surface area released after applying more or less intense ultrasonic treatment to the powder previously dispersed in ethanol ( Rajaonarivony *et al*., 2019). A similar methodology was applied here to obtain global values for the soft and hard agglomerates. Soft agglomeration was evaluated from specific surface area measured after applying ultrasonic treatment with the internal granulometer probe, and hard agglomeration was evaluated from specific surface area measured after applying a more powerful treatment with an external ultrasonic probe.

**Figure 7.**
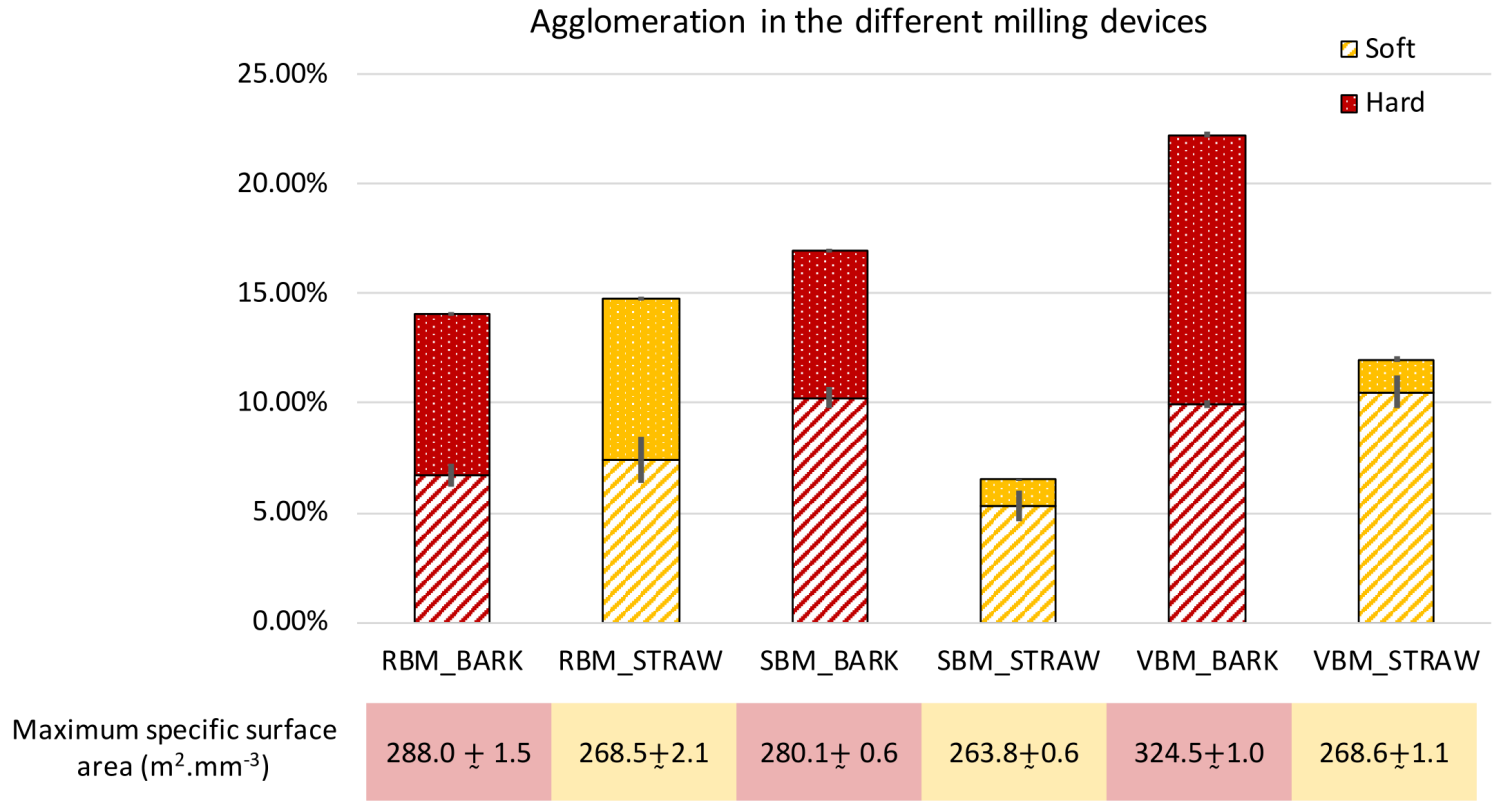
Extent of soft and hard agglomeration observed in RBM, SBM and VBM powders. Extent of soft and hard agglomeration observed in RBM (rotary ball mill), SBM (stirred ball mill) and VBM (vibratory ball mill) powders and maximum developed specific surface area of the different powders.

**Figure 8.**
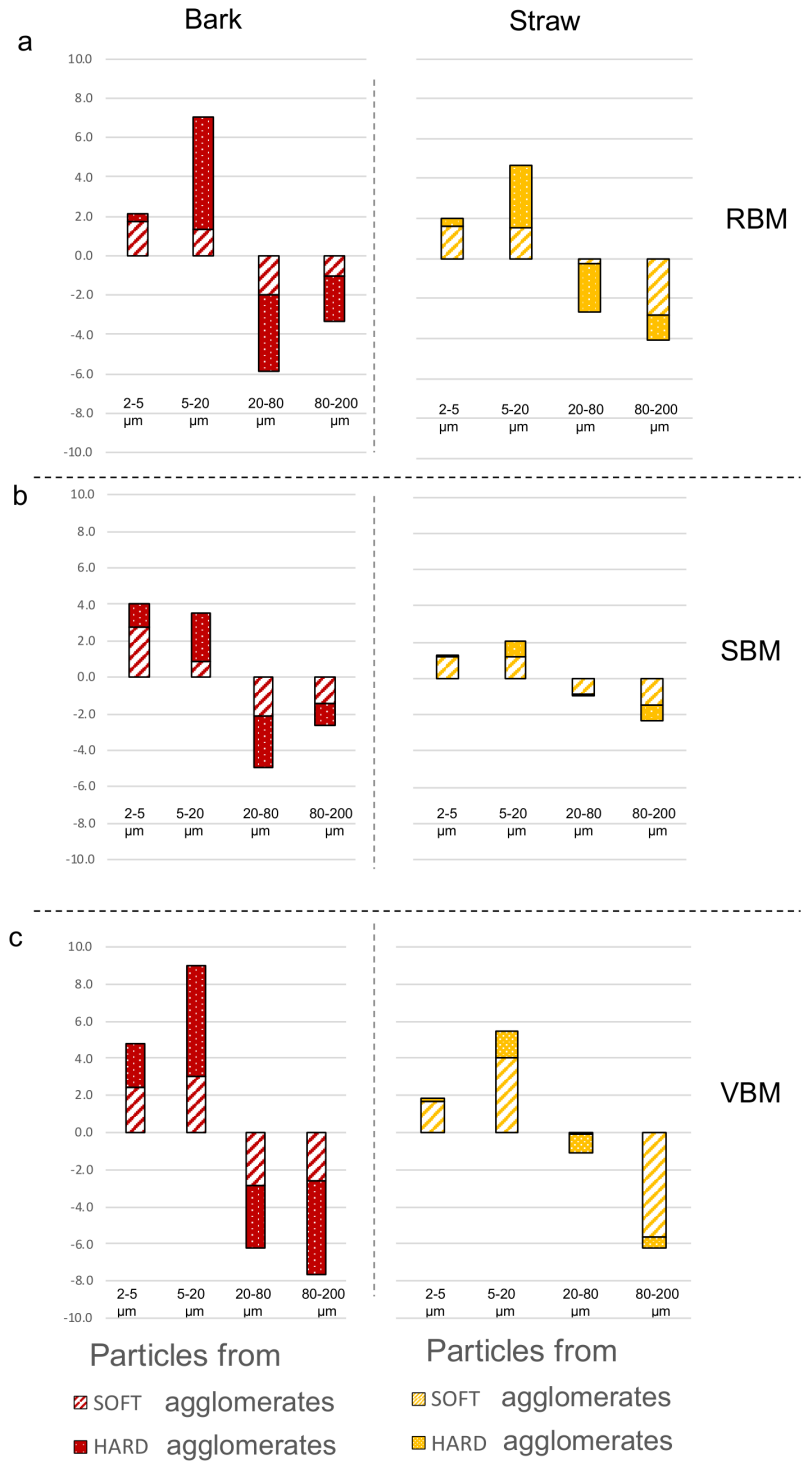
Soft and hard agglomeration status in four particle size-classes for bark and straw powders. Soft and hard agglomeration status deduced from the changes in specific surface areas following mild and strong ultrasonic treatments in four particle size-classes for bark (red) and straw (yellow) powders from the ( **a**) RBM (rotary ball mill), ( **b**) SBM (stirred ball mill) and ( **c**) VBM (vibratory ball mill).

Figure 7 reports the results obtained as well as the maximum specific area obtained after applying the intense ultrasonic treatment with the external probe. To evaluate the sizes of the agglomerates and their component particles, we employed an approach inspired by population balance modelling ( Gil *et al*., 2015) to study the evolution of the volume weight of different population classes when applying the two ultrasonic treatments. Figure 8 reports the results for each milling device and for both biomasses.

Total agglomeration (soft + hard) varied according to milling device. Globally, it was stronger for bark (at least 14%) than for straw (less than 15% at most). This difference may be explained by the chemical composition of bark, which is rich in polyphenols ( Catarina *et al*., 2019) that create numerous strong inter-particle bonds.

In the case of bark, the total agglomeration in the VBM was greater than 20%, which means that more than ⅕ of the surface of the powder is not directly accessible. However, the VBM device was able to achieve a 20% greater total specific surface area than the RBM and the SBM due to the erosion of coarser particles under the attrition force. These fines would tend to clump together, thus strengthening the agglomeration, but the additional surface created by the VBM nevertheless remains trapped inside intensely-bound hard agglomerates (which account for more than 50% of the total agglomeration). As the PSD profiles ( Figure 2) do not show significant differences in span and d10, we assume that the very fine particles stick to coarser ones and agglomerate as soon as they are produced by the comminution process.

For the bark milled with the RBM, most of the agglomerates are sized between 20–80 µm and composed of 5–20 µm-class particles ( Figure 8). In the SBM, most of the agglomerates are also sized between 20–80 µm but are composed of almost equal proportions of 2–5 µm-class and 5–20 µm-class particles. In contrast, VBM agglomerates (especially the hard agglomerates) have proportionately bigger sizes (between 80–200 µm) and are composed of ⅓ 2–5 µm particles and ⅔ 5–20 µm particles. This suggests that the 5–20-µm particles may have been coated by the smallest ones (2–5 µm) or that the finest particles act as a cement between larger ones. In both the SBM and VBM mills, the variations of the 2–5 µm classes are more than two times those observed in RBM, at 4 and 5 against 2, respectively. This confirms the previous assumption that the agglomeration may be related to the emergence of fine particles during the comminution process that agglomerate as soon as they are produced. 

For the straw powders, the maximum specific surface areas were similar between all the milling devices, and the powders showed a lower extent of agglomeration than for the bark. PSD analysis showed that straw powders contained smaller particles than bark powders, especially in the SBM and VBM processes, and had a wider span, suggesting that in the case of straw feed material, not all the fine particles produced during the comminution process are involved in the formation of agglomerates. Furthermore, and conversely to bark, straw led to significantly less hard agglomeration than soft agglomeration in the SBM and VBM, whereas both these milling devices generated a large amount of fine particles. Figure 8 shows that very few particles in the 2–5 µm size class were involved in hard agglomerates. The RBM device appeared to produce a higher amount of hard agglomeration. This could be explained by the longer time of the RBM comminution process for straw (23h) than for bark (4.5h; Table 1). In general, for the straw, whatever the device, there were more agglomerates in the 80–200 µm size range than in the 20–80 µm size range. These agglomerates were mainly composed of particles originating from the 5–20-µm size class.

Interestingly, straw particles milled in the SBM led to very little agglomeration. The dual mechanical forces (impact and attrition) in the SBM thus appear well adapted to limit the agglomeration of straw particles. 

### Flow properties of the powders

The flow properties of the straw and bark powders ground in the three ball mills were assessed by measuring their compressibility and cohesion. The results are reported in Table 2.

**Table 2.** Particle shape factors of of IM_bark and IM_straw powders. Median equivalent spherical diameter, elongation and convexity of the feeding powders: IM_bark and IM_straw.

	Median equivalent spherical diameter	Median elongation	Median convexity
IM_bark	76.5 μm	0.33	0.85
IM_straw	235.5 μm	0.55	0.84

***Compressibility.*** Compressibility was obtained using two approaches: first, as the difference between bulk and tapped densities used to calculate the Carr index (Ci), and second, as the reduction in volume under a 15 kPa normal constraint in the FT4 device (Cp).

The tapped measurements for all the powders fell into roughly the same range. According to the Carr table ( Carr, 1965), all the products were categorized as powders with poor/slightly poor flowability, but the table does not help to give a sharper classification. However, our values show that straw is slightly more compressible than bark. Straw and bark powders from the RBM and VBM had a similar Carr index, whereas SBM bark powder had the lowest Carr index and SBM straw powder the highest Carr index.

The measures in the FT4 device evidenced larger differences in compressibility (Cp) between biomass samples. On average, the Cp values were 35% lower for bark powders than straw powders, whereas there are only slight differences between bark and straw samples between the different milling devices. Note, however, that VBM straw had the highest overall compressibility, and it also had by far the highest elongation value and the widest span. It is well known that the compressibility of a powder results from the packing possibilities of its particles within the powder bed ( Pachón-Morales *et al*., 2019). Bark particles were less stretched and less dispersed than straw particles, and thus logically gave less compressible powders. On the other hand, elongated straw particles can create more voids inside the powder bed due to their random stacking, with more re-arrangement possibilities under compression. Within the whole sample set, we found that compressibility was well correlated with the elongation of particles >20µm (r ^2^=0.85) and with span (r ^2^=0.77). Moreover, it is possible that long straw particles can readily deform under the normal constraint, and thus add compressibility, in contrast with the solid bark particles. The influence of type of comminution mechanism on compressibility of the powders therefore operates mainly through the geometric characteristics imparted to the particles, namely shape of the large particles and PSD.

In other words, the VBM which works mostly by attrition, erodes the fibres into large elongated particles accompanied by a fine dust, giving a more compressible product than the RBM and SBM. However, this is only valid for the fibrous straw biomass. Indeed, with the layered bark biomass, the differences in span and particle shapes are too small to have a significant influence on the compressibility of the final powder. 

***Cohesion.*** Cohesion can be seen as the minimum constraint value needed to put an unconsolidated powder bed into flow. It translates the effect of the inter-particle forces which have a tendency to stick the particles together in the powder bed and generate resistance to deformation and flow. Higher values mean more cohesive powders. Table 3 reports the cohesion values (Co) obtained from the regression of breakage constraint to consolidation constraint of the powders between 1 kPa and 2.5 kPa.

**Table 3.** Rheological properties of bark and straw powders obtained from the RBM (rotary ball mill), SBM (stirred ball mill) and VBM (vibratory ball mill).

		Carr index (Ci) (%)	FT4 – 15 kPa compressibility (Cp) (%)	FT4 cohesion (Co) (kPa)
RBM	bark straw	27.3 27.0	21.0 27.0	0.432 0.398
SBM	bark straw	24.3 30.0	17.0 29.0	0.490 0.384
VBM	bark straw	27.6 27.2	18.0 30.0	0.600 0.431

On average, bark powders were 20% more cohesive than straw powders. The intensity of the inter-particle forces increases with decreasing particle sizes, as particles get closer together ( Gnagne *et al*., 2017). However, we found no correlation between the cohesion of the powders and their PSD and shape characteristics (d10, d90, span, elongation) in our sample set because they exhibit almost no difference between the different particle size/shape indicators. In principle, spherical particles offer fewer potential contact points than elongated particles, which should minimize inter-particle forces ( Pachón-Morales *et al*., 2019). However, here, bark particles had less elongated shapes but higher cohesion. Therefore, the physical and chemical surface quality of the particles may play a key role in establishing interactions. As convexity, which accounts for particle surface roughness, was not very different in the two types of biomass samples (7% difference on average between bark and straw), we conclude that the strength of inter-particle bonds is largely dictated by chemical composition of the particle surface. During milling, chemical groups are revealed as new surfaces get exposed and others get oxidized, thus continuously modifying surface reactivity and therefore inter-particle interactions. Pine bark has a higher reactive phenolic compounds content than straw. Also, unlike bark, straw contains waxes (~1% mass content) ( Hamilton, 1995) that are mainly localized in a smooth epidermis tissue ( Valdez-Vazquez *et al*., 2017). Wax-rich particles dispersed by the milling process may play a lubricating role, which could explain in part the lower cohesion (Co) and higher compressibility (Cp) of straw powders by facilitating sliding and repulsion between particles.

Interestingly, there was a strong correlation between powder cohesion and particle agglomeration in the whole set of samples (r ^2^ = 0.9) and an even stronger correlation when considering only bark samples (r ^2^ = 0.99). This correlation is not evidence of a causal link between the two properties but rather a common cause that can be found in the surface composition of the powders. The particle surface composition allows more or less strong bonding of the fine particles under the mechanical force imparted either in the mills for agglomeration or in the FT4 device for cohesion.

Concerning the effects of the different mills, RBM and SBM yielded powders with fairly similar cohesion values, while the VBM clearly produced the most cohesive powders, both with bark and straw. Since no direct significant correlation was found between cohesion and any single measured value of particle characteristics, it is likely that the influence of mill type was driven by a combination of slight variations imposed on powder characteristics in relation to the main mechanical force, such as production of fines, shapes of the different populations of particles, physical surface status, and degree of surface oxidation.

### Discussion on the properties of the powders in relation to type of ball mill

All three ball- mills used in this study achieved the target mean particle size of 20 µm for both pine bark and wheat straw powders. However, apart from this single common trait, all the powders generated differed in several particle characteristics and flow properties.

**Figure 9.**
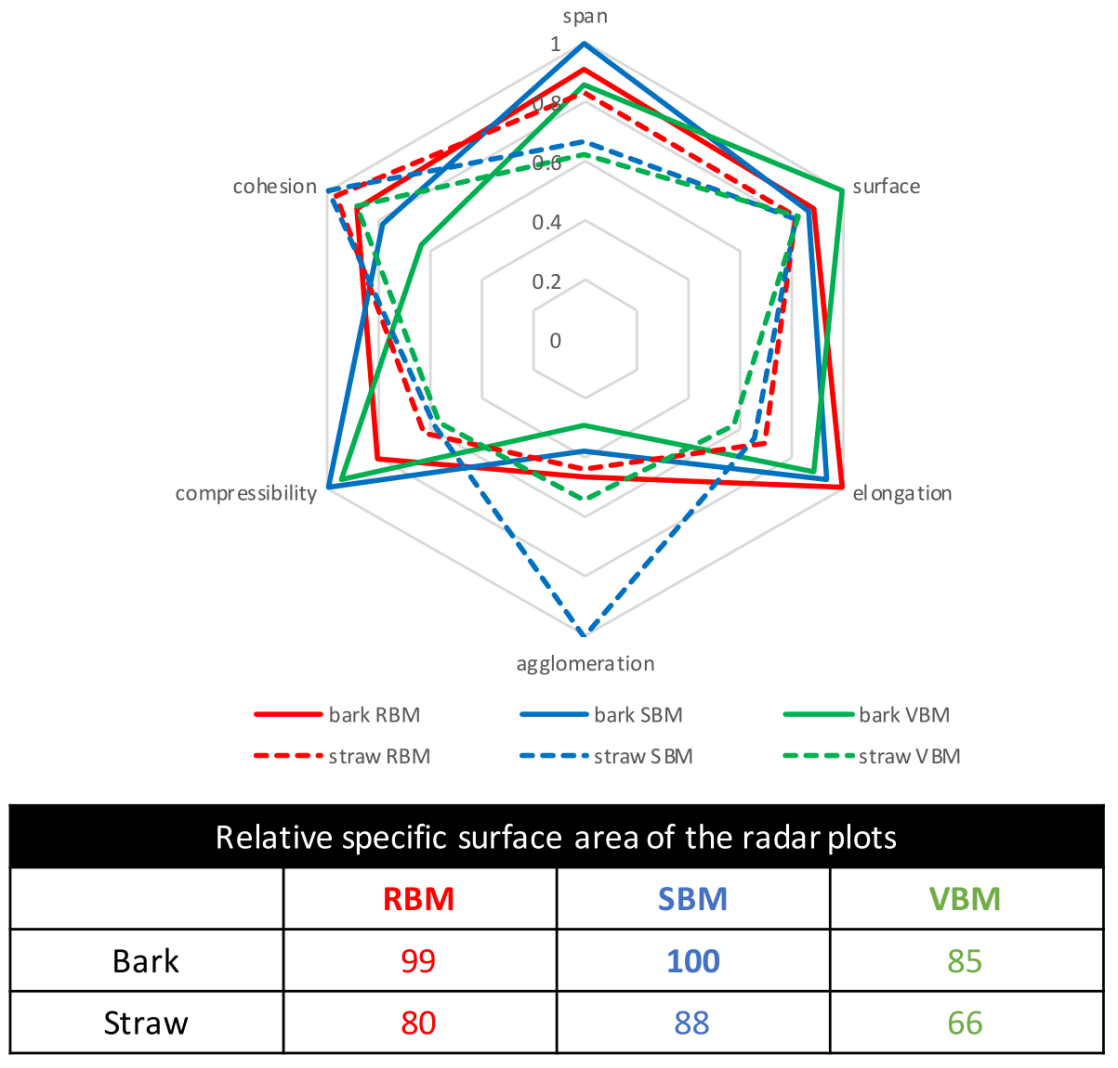
Comparison of the bark and straw powders properties generated by the three ball mills. Comparison of the bark and straw powders properties generated by the three ball mills in terms of particle and flow properties and relative areas of the radar plots (as % of the largest area corresponding to the best sample). For each indicator, a value of 1 corresponds to the best behaviour for the property considered among the whole set of all powders.

***Quality of the powders.*** The quality of a powder can be described by a set of indicators corresponding to ‘good’ and predictable properties. In this study, good powder properties would mean low span, high specific surface, low elongation, little agglomeration, low compressibility and weak cohesion. We made a standardized comparison of the six powders produced by the three mills, considering that the best sample should exhibit maximum particle surface area and minimum span, elongation, agglomeration, compressibility and cohesion. Therefore, to get a comprehensive representation of the data, the indicators we used were the inverse of the span, elongation, agglomeration, compressibility and cohesion values, normalized by the maximum value recorded among the whole powder dataset. For each indicator, the powder demonstrating the best behaviour for that property was given a value of 1. The results are presented as a radar plot in Figure 9.

In this comparative analysis, the weighting given to each criterion is the same. The powders could be assigned different criterion weightings to favour properties required in the specific target applications.

In general, for both biomasses, RBM and SBM yielded powders with equivalent properties, whereas VBM powders were clearly less good. More specifically, for bark, RBM powder had the advantage of little total agglomeration and cohesion, whereas SBM powder had the advantage of narrow span and low compressibility. The VBM powder, although exhibiting a high specific surface area, was penalized by high agglomeration and cohesion. For straw, SBM powder was the best due to a very little total agglomeration, RBM powder was good due to its narrow span, and VBM powder was the worst due to poor span, elongation and agglomeration properties.

In an overall comparison, the relative area of the radar plots (expressed as percent of the highest radar area) can be used to rank the different powders as it serves as an average assessment of all-round quality. The two best-quality powders were SBM_bark and RBM_bark, followed by SBM_straw, then VBM_bark. VBM_straw powder was the worst-quality powder due to its poor span and elongation properties responsible for high compressibility. In conclusion, in terms of powder quality, pine bark is a better starting material for fine comminution than the fibrous wheat straw, and the mills that work by impact (RBM) and impact + attrition (SBM) yield powders with better properties than the mill that works by attrition (VBM) on the same starting material.

**Figure 10.**
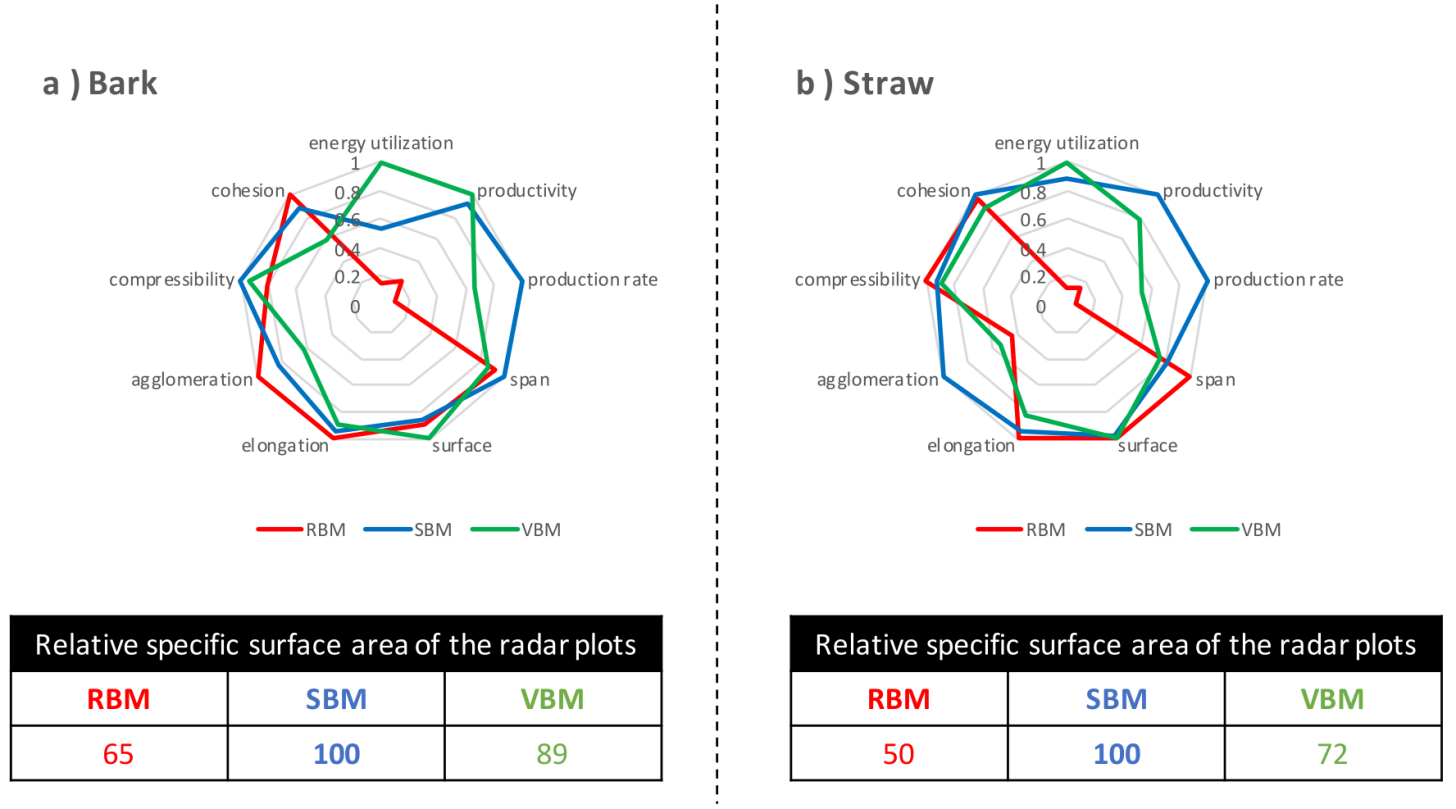
Processing quality of bark and straw powders produced by the three ball mills. Comparison of the processing quality of ( **a**) bark and ( **b**) straw powders produced by the RBM (rotary ball mill), SBM (stirred ball mill) and VBM (vibratory ball mill) including process efficiency indicators and powder quality indicators. Relative areas of the radar plots (expressed as % of the largest area, i.e. the best sample) were calculated separately for bark and straw.

***Overall performance of ultrafine milling.*** Finally, we compared the powders by factoring in the quality of the powders produced and the process efficiency of the mills used to produce them. The process efficiency data came from the previous study of Rajaonarivony *et al*. ( Rajaonarivony *et al*., 2021) where they assessed the energy performances of the three ball mills when micronizing bark and straw materials to 20 µm-centred powders. Rajaonarivony *et al*. used three indicators as markers of milling efficiency: energy utilization (m ^2^. kWh ^-1^), productivity (kg. kWh ^-1^), and surface production rate (m ^2^. g ^-1^.h ^-1^). These indicators, also normalized using the highest values for each biomass, were added to the powder quality indicators in radar plots ( Figure 10) in order to compare the combination of process efficiency and product quality for each milling device.

For both biomasses, the VBM was the best in terms of energy utilization and the SBM was the best in terms of productivity and processing rate. The RBM was strongly penalized by its excessively long processing time resulting in low productivity and very poor energy efficiency. Overall, for each biomass across the whole set of process and product quality indicators, SBM ranked first based on the relative areas of the radar plots. The SBM mill was proportionately more efficient with straw than with bark compared to the two other mills, and therefore appears to be well adapted for the comminution of resistant fibrous straw-type substrates (e.g. stems, stalks, bagasse, husks). The VBM, although the best option in terms of energy use, had a lower total score (i.e. relative specific surface area of the radar plots) due to poor powder properties, in particular with straw. RBM was the least suitable option, especially in the case of straw.

It should be borne in mind that the three ball mills tested here were operated in batch processes, with a share of the particles remaining repeatedly submitted to the mechanical force even though they have met the specification in terms of targeted size. This share of the powders is likely to promote agglomeration and induce unnecessary energy consumption. Using continuous-process mills with automatic selection and recovery of target-sized product would modify both the energy efficiency of comminution and the quality of the powders, with the same mechanical forces and biomass types used in this study.

## Conclusion

Here we compared the properties of powders produced by extensive dry milling of lignocellulosic biomass after processing pine bark and wheat straw in three different ball mills. A rotary ball mill (RBM), a vibratory ball mill (VBM), and a stirred ball mill (SBM) reduced the lignocellulosic material into 20 µm-average-particle-size powders by using predominantly impact (RBM), attrition (VBM) and impact + attrition (SBM) mechanical forces. The VBM yielded powders with large elongated particles mixed with high amounts of fines, as it acted mostly by erosion. The VBM powders also exhibited more agglomeration due to the large abundance of fines. The RBM yielded powders that had more evenly distributed particle sizes and shapes and less agglomeration due to a preferential cross-fragmentation mechanism. The SBM yielded powders with intermediate characteristics in terms of particle shape and agglomeration together with low compressibility and cohesion as a result of mechanical force combining fragmentation and erosion. Our analyses of the two tested biomasses found that pine bark is more easily grindable than the fibrous wheat straw *.* The pine bark powders were less dispersed, less compressible but much more cohesive than the straw powders, in relation with the densities, reactive chemical surface composition and more compact shapes of the particles. In summary, taking into account the energy-use and production-rate performances of the mills ( Rajaonarivony *et al*., 2021) together with the quality of the powders produced, an SBM-type mill appears to be the best option for efficiently micronizing lignocellulosics, due to the complementarity of the impact and attrition forces it delivers.

## Data availability

## Underlying data

Data INRAE: Properties of biomass powders resulting from the fine comminution of straw and bark feedstocks by three types of ball-mill set-up (RBM, SBM, VBM). https://doi.org/10.15454/F9ZSBS ( Mayer-Laigle *et al*., 2021b).

This project contains the following underlying data:

- Bark_Straw_MEB (raw SEM pictures of bark and Straw ground powders)
- Size and agglomeration (agglomeration and particle size distribution of bark and straw powders)
- Rheology (Carr index, compressibility and cohesion test for bark and straw powders)
- Shape (convexity and elongation of bark and straw powders ground with the impact mill and in the three ball mill devices)

Data are available under the terms of the Licence Ouverte (Open Licence) Version 2.0.
