# Supplementary material for: Properties of biomass powders resulting from the fine comminution of lignocellulosic feedstocks by three types of ball-mill set-up
Source: Open Res Eur. 2022 Mar 16;1:125. Originally published 2021 Oct 19. [Version 2] doi: 10.12688/openreseurope.14017.2 (PMC10445875; doi:10.12688/openreseurope.14017.2)
Supplement: Peer review history

# Peer review: Reviewer response for version 1

**DOI:** 10.21956/openreseurope.15105.r27952

**Published:** 2021-12-15

**Authors:** Christine Frances

**Affiliations:** 1 Laboratoire de Génie Chimique, CNRS, INPT, UPS, Université de Toulouse, Toulouse, France

This paper presents experimental results obtained from milling runs carried out on two types of lignocellulosic materials and using three different mills. The systematic and rigorous analysis of the properties of the milled products (in terms of particle size, fragment shape, ability to form agglomerates and powder flow properties) has clearly shown the impact of the main mode of action in these different mills on the properties of the biomass. In order to produce fragments with a median diameter of 20 µm, the operating conditions (process parameters, mass of the sample in the mill chamber, duration of the batch operation, etc.) under which the tests were carried out are very different depending on the type of mill. So the statistical analysis proposed at the end of the paper by introducing indicators relating to energy efficiency in addition to the criteria relating to the quality of the powder is highly relevant.   

 Here are some comments and minor points:

- eq.2 : since the particle size is characterized by their diameter, the use of the average diameter instead of the radius of particles could be more relevant.
- In the paragraph above eq. 5, the notation used for the volume weight of particle populations is not the same as in the equation.
- p.6,in the paragraph below eq. 7, I think the comment on the meaning of the convexity factor is a bit confusing, instead of fractal geometry I would have said that the convexity factor gives an indication of the surface roughness, as it is explained on p. 9 (in the paragraph on particle shape).
- p.9 related to the comment “median equivalent spherical diameter values deviated by about 20% from the values measured by laser diffraction …”. I think another explanation could be that the laser diffraction size measurement is based on the volume, whereas image analysis characterises the size of particles on the basis of the number.
- figures 4 and 5. The minimum limit for the particle size is to be harmonised (2 or 2.5 µm) between the legends on the graphs and the titles of the figures.
- p. 15 and p.16. The capital letter R (as in R ^2^=0.99) is more often used to designate the correlation coefficient.

Is the study design appropriate and does the work have academic merit?

Yes

Is the work clearly and accurately presented and does it cite the current literature?

Yes

If applicable, is the statistical analysis and its interpretation appropriate?

Yes

Are all the source data underlying the results available to ensure full reproducibility?

Yes

Are the conclusions drawn adequately supported by the results?

Yes

Are sufficient details of methods and analysis provided to allow replication by others?

Yes

Reviewer Expertise:

Chemical engineering on particulate processes

I confirm that I have read this submission and believe that I have an appropriate level of expertise to confirm that it is of an acceptable scientific standard.

# Response

**Published:** 2022-02-21

**Authors:** Claire Mayer-Laigle

**Affiliations:** INRAe, France

Thank you for your interest in our work and for your comments which were helpful to correct the minors remaining errors.

# Peer review: Reviewer response for version 1

**DOI:** 10.21956/openreseurope.15105.r27852

**Published:** 2021-11-03

**Authors:** Peter. J. Deuss

**Affiliations:** 1 Department of Chemical Engineering, University of Groningen, Groningen, The Netherlands

The Manuscript by Rajaonarivony *et al *contains a very interesting study into the powders from pine bark and wheat straw produced by different milling setups. The analysis of the powders is thorough and an in-depth comparison is provided of the powders based on the biomass as well as the type of mill. This demonstrates that choice of equipment matched with particular biomass can have significant effects on the powder in terms of shape, coarseness and agglomeration behavior. This can be important in terms of valorization of biomass residue streams towards biobased products.

 Only two small comments:

- On Page 12 there is a reference to figure 6j for VMB particles. I guess this should be figure 6i?
- On page 13 Maybe difference in particle interactions between bark and straw is not only due to polyphenols. The bark also contains more "fatty" compounds and terpenoid. These might also cause stronger interaction. 

Is the study design appropriate and does the work have academic merit?

Yes

Is the work clearly and accurately presented and does it cite the current literature?

Yes

If applicable, is the statistical analysis and its interpretation appropriate?

I cannot comment. A qualified statistician is required.

Are all the source data underlying the results available to ensure full reproducibility?

Yes

Are the conclusions drawn adequately supported by the results?

Yes

Are sufficient details of methods and analysis provided to allow replication by others?

Yes

Reviewer Expertise:

Biomass Processing

I confirm that I have read this submission and believe that I have an appropriate level of expertise to confirm that it is of an acceptable scientific standard.
